# Estimating the relative importance of epidemiological and behavioural parameters for epidemic mpox transmission: a modelling study

**DOI:** 10.1186/s12916-024-03515-8

**Published:** 2024-07-18

**Authors:** Madhav Chaturvedi, Isti Rodiah, Mirjam Kretzschmar, Stefan Scholz, Berit Lange, André Karch, Veronika K. Jaeger

**Affiliations:** 1 Institute of Epidemiology and Social Medicine, University of Münster, Münster, Germany; 2 Department of Epidemiology, Helmholtz Centre for Infection Research, Brunswick, Germany; 3 Department of Epidemiology, University Medical Center Utrecht, Utrecht, the Netherlands; 4 Martin-Luther-University Halle-Wittenberg, Halle, Germany; 5 German Center for Infection Research, Brunswick, Germany

**Keywords:** Infectious disease modelling, Mpox, Global sensitivity analysis

## Abstract

**Background:**

Many European countries experienced outbreaks of mpox in 2022, and there was an mpox outbreak in 2023 in the Democratic Republic of Congo. There were many apparent differences between these outbreaks and previous outbreaks of mpox; the recent outbreaks were observed in men who have sex with men after sexual encounters at common events, whereas earlier outbreaks were observed in a wider population with no identifiable link to sexual contacts. These apparent differences meant that data from previous outbreaks could not reliably be used to parametrise infectious disease models during the 2022 and 2023 mpox outbreaks, and modelling efforts were hampered by uncertainty around key transmission and immunity parameters.

**Methods:**

We developed a stochastic, discrete-time metapopulation model for mpox that allowed for sexual and non-sexual transmission and the implementation of non-pharmaceutical interventions, specifically contact tracing and pre- and post-exposure vaccinations. We calibrated the model to case data from Berlin and used Sobol sensitivity analysis to identify parameters that mpox transmission is especially sensitive to. We also briefly analysed the sensitivity of the effectiveness of non-pharmaceutical interventions to various efficacy parameters.

**Results:**

We found that variance in the transmission probabilities due to both sexual and non-sexual transmission had a large effect on mpox transmission in the model, as did the level of immunity to mpox conferred by a previous smallpox vaccination. Furthermore, variance in the number of pre-exposure vaccinations offered was the dominant contributor to variance in mpox dynamics in men who have sex with men. If pre-exposure vaccinations were not available, both the accuracy and timeliness of contact tracing had a large impact on mpox transmission in the model.

**Conclusions:**

Our results are valuable for guiding epidemiological studies for parameter ascertainment and identifying key factors for success of non-pharmaceutical interventions.

**Supplementary Information:**

The online version contains supplementary material available at 10.1186/s12916-024-03515-8.

## Background

Mpox, formerly known as monkeypox [1], is a zoonosis caused by an orthopoxvirus which results in a smallpox-like disease in humans. Mpox was first discovered in humans in 1970 in Central and Western Africa [2]. Later, infections were also observed on other continents—the largest outbreak hitherto occurred in the United States in 2003, where 47 confirmed and probable cases were identified, all having contracted the disease from animals [3]. Other infections in non-endemic regions have occurred since then, usually associated with travel [4–6]. There is evidence to show that smallpox vaccination grants some level of immunity against the mpox virus [7]; in recent decades, an increase in both the number of affected areas and the number of detected cases in such areas has been noted, corresponding to declining population immunity after the eradication of smallpox and thus the cessation of smallpox vaccination. Against this background, recent mathematical modelling studies revealed clear evidence of the potential for further mpox outbreaks [8].

This epidemic potential was realised in 2022, which saw outbreaks in several countries where mpox was previously non-endemic. These outbreaks were distinct in that the disease predominantly spread in and affected men who have sex with men (MSM) [9–11]. Modelling studies have since shown that a heavy-tailed distribution of sexual contacts, with a subgroup of the MSM population having many more sexual partners than the mean (sometimes called the ‘core group’), could sustain an mpox outbreak based on sexually associated contact transmission alone [12]. More recently, there has also been evidence of potential sexual contact transmission during the 2023 outbreak in the Democratic Republic of Congo [13]. However, previous evidence [14] and a few confirmed cases in children during the recent outbreaks [15] suggest that the virus is not transmitted solely through contacts associated with sexual activity, and more general forms of close or physical contact can also be means of human-to-human transmission. Theoretically, then, the virus should also find it easy to spread in other settings with a large number of physical contacts, like care homes and kindergartens, daycare centres, or other such childcare and education institutions.

That the majority of detected infections during the recent outbreaks have been in the MSM community and barely any have been in children, care home residents and such [9–11] could imply that the probability of transmission during sexually associated physical contacts is much higher than that during other forms of physical contact. However, epidemiological studies quantifying these parameters are scarce, given the private and sometimes anonymous nature of sexual contacts, especially in the core group [16], as well as the lack of substantial transmission in other settings. Uncertainty also surrounds the quantification of the immunity to mpox conferred by the smallpox vaccine in different age groups dependent on time since vaccination, and the proportion of the population that has had the smallpox vaccination in the first place, although in theory ascertaining these parameters should be an easier task.

Faced with these uncertainties, efforts to model the recent outbreak and proposed or implemented interventions have been limited by the need to make many assumptions about key parameters or consider a very wide range of scenarios [12, 17–19]. In this paper, we present a compartmental infection dynamics model that can simulate transmission in different contact networks/settings and also model contact tracing that can take into account realistic delays and contact patterns, without the added computational and conceptual complexities of a network model or an agent-based model. We use this model to determine which epidemiological and behavioural parameters the transmission of mpox is most sensitive to, with a focus on infections in the MSM community and other high physical-contact contexts like kindergartens and care homes. We believe that this information can be used to help guide the prioritisation of resources in epidemiological studies to the most impactful parameters and can also help infectious disease modelling efforts ascertain which parameters can be safely fixed at assumed values and which parameters call for more scrutiny. We also investigate the efficacy of population-level interventions at controlling the spread of mpox and how sensitive this efficacy is to various efficiency or accuracy parameters related to the intervention. This can help guide improvements in certain aspects of non-pharmaceutical interventions and is thus useful for future outbreak preparedness.

## Methods

### Model

Our model is a compartmental, discrete-time, stochastic model that describes the epidemiological dynamics of mpox infection in an S-E-I-R (Susceptible-Exposed-Infectious-Recovered) framework, with additional compartments to model quarantine and isolation of individuals, and the presence of asymptomatic or otherwise undetectable infection. In the main text, we present only the main features of the model; a complete and formal definition is supplied in Supplementary File 1 [20–38]. The model was built using the odin.dust package (version 0.3.9) [39] in R 4.3.1 [40]. We used evidence from previous studies to inform the model structure when possible. However, given the recency of the outbreaks and the complexity of our model especially with regard to the metapopulation structure, it was not always possible to find relevant studies to base model structure on, and we made educated assumptions in these cases.

#### Metapopulations

The population in the simulation is stratified into 12 age groups (0–4, 5–9, 10–14, 15–17, 18–24, 25–34, 35–44, 45–54, 55–64, 65–69, 70–74, 75 +). A proportion of the youngest age group (0–4 years old) is assumed to attend a kindergarten, daycare, or other such institution, and a proportion of the population above the age of 65 is assumed to consist of care home residents. Some individuals in the age groups that comprise the 18–65 band are assumed to be teaching staff in kindergartens/daycares, and some are carers in care homes. Furthermore, a proportion of the 18–74-year-olds (in each setting except care home residents) are men who have sex with men (MSM), and the MSM subpopulation is further divided into high and low sexual activity groups.

We have made efforts to ensure that the distribution of the population into these metapopulations is similar to the real distribution thereof in the population of Berlin, Germany. However, the numbers are estimates from various data sources (described in Supplementary File 1) and some inconsistencies needed to be resolved based on assumptions.

#### Transmission routes and course of infection

We assumed that the mpox virus can be transmitted through any sort of close, physical contact, but has a much higher probability of transmission through contacts which include a sexual component. Therefore, the model allows for four routes of transmission: through sexual contacts; household contacts; contacts between children and teachers and children and children in kindergarten settings; and contacts between residents and carers and residents and residents in care home settings.

The pre-infectious period of the virus is assumed to be the same as the incubation period, variable, and follow a log-normal distribution based on previous evidence [22]. Variability in the pre-infectious period is modelled by having sub-compartments of the exposed compartment that track the number of days since the start of the incubation period and then assigning probabilities of transitioning to the infectious stage for each day of the pre-infectious period.

The infectious period is also assumed to be variable and is modelled using a gamma distribution as a more realistic alternative to the often implicitly used exponential distribution [41], with transitions to the Recovered compartment modelled similar to transitions from the Exposed to Infectious compartments. The Infectious compartment is split up into ‘Detectable’ and ‘Undetectable’ compartments. Undetectable infectious individuals go through the infectious period and recover and can transmit infection to others throughout the infectious period. Detectable infectious individuals have a probability of being detected on each day of the infectious period bar the first two, although they may recover without ever being detected. On detection, these individuals isolate and do not have further contact (in the sense of physical contact) or infect anyone else.

#### Existing immunity

We assumed an all-or-nothing model for the immunity to mpox conferred by the smallpox vaccination, i.e. we assumed that a proportion of individuals who have had the smallpox vaccination in the past are completely immune (in which case we move them to the Recovered compartment at the start of simulation), and the rest are equally as susceptible to mpox as those without smallpox vaccinations. The proportion of people who have such immunity depends on both the proportion of people in each age group who were vaccinated and the level of protection provided by a smallpox vaccination.

We expect that the proportion of people who were vaccinated against smallpox would be higher in the older age groups than the younger age groups, as smallpox vaccination was slowly phased out as the world got closer to eradication. However, the vaccine may provide more immunity in the younger age groups, if the immunity provided this way wanes quickly. Therefore, the proportion of individuals in the entire population that have immunity to mpox due to a smallpox vaccination is potentially a complex, two-parameter function of age. In the analyses, we assumed that immunity conferred this way does not wane and that the proportion of individuals vaccinated against smallpox decreases linearly from 90% in the oldest age group to 40% in the youngest age group old enough to have been alive during smallpox vaccination drives in Germany (the 35–45 years old group). The model parameter governing immunity can then be interpreted as the level of immunity to mpox conferred by the smallpox vaccination.

#### Non-pharmaceutical interventions

The non-pharmaceutical interventions (NPIs) employed in Germany to combat the 2022 mpox outbreaks were a combination of contact tracing, targeted pre-exposure vaccinations for those deemed to be at high risk, and post-exposure vaccinations of traced contacts (ring vaccinations). We consider the same NPIs in our model.

In the model, susceptible and exposed individuals are traced with a probability dependent on when they were last contacted by an infectious, detectable individual, and a base tracing probability that represents parameters like tracing accuracy, adherence to tracing regulations, etc. There is also a lag in tracing, assuming that there will be a delay between a case being detected and their contacts being traced. Furthermore, all traced contacts are vaccinated if ring vaccinations are active, but the proportion successfully immunised is governed by a ‘vaccination efficacy’ parameter, not to be confused with the smallpox vaccination parameter that determines how many individuals had *pre-existing* immunity due to the smallpox vaccination.

To model pre-exposure vaccinations, we assume that a number of vaccinations are offered to the ‘high risk’ group (defined in this instance by the Robert Koch Institute as individuals in the MSM community that have a high frequency of sexual contacts [42], mirrored in the model as the high sexual activity subgroup of the MSM metapopulation) every day, and successful immunisation is governed by the same ‘vaccination efficacy’ parameter as in ring vaccinations.

In line with the Robert Koch Institute guidelines [20], we assume that this vaccination can also successfully immunise exposed individuals who are in the first 4 days of their incubation period.

### Calibration

We fit the model to data about reported cases of mpox per calendar week (for calendar weeks 21–45) in Berlin, obtained from the Robert Koch institute database SurvStat [43]. We initialised our model at the start of calendar week 19 and assumed that the observed data would be Poisson-distributed around the cases detected in a week in the model (with a small amount of noise added to avoid zero expectation). We used the R package mcstate version 0.9.18 [44] to run a particle Markov chain Monte Carlo (pMCMC) algorithm and took means of the samples generated as the fitted parameter values. Comprehensive details about the pMCMC run are given in Supplementary File 2 [43–45]. Table 1 shows the parameters included in the fitting process, along with bounds on their values. During the 2022 outbreaks, the smallpox vaccine IMVANEX—previously used in smallpox immunisation drives—was used for pre- and post-exposure vaccinations against mpox in Germany [20]. Therefore, for calibration, the same parameter governs both immunity due to previous smallpox vaccination and immunity conferred by vaccination administered as part of non-pharmaceutical interventions.

**Table 1:** Parameters that were calibrated using case data from Berlin, and the bounds within which they were calibrated

Parameter to be fitted	Bounds
Probability of transmission per non-sexual contact	(0.0, 0.1)
Probability of transmission per sexual contact	(0.0, 0.5)
Proportion of infections that are detectable	(0.5, 1.0)
Initial number of infections (per age group in which infections initialised) (integer)	(0, ∞)
Ratio of sexual contacts of core MSM group to rest of MSM population	(10.0, 30.0)
Assortativity in sexual mixing between core group and rest of MSM population	(0.5, 1.0)
Probability that vaccination confers immunity*	(0.5, 1.0)
Base probability that a contact of a detected case will be traced	(0.5, 1.0)

^*^The same vaccination parameter governs immunity due to a previous smallpox vaccination and immunity due to pre- and post-exposure vaccinations as part of non-pharmaceutical interventions

We assumed a lag in tracing of 2 days and an average of 200 daily pre-exposure vaccinations offered.

### Analyses

We used the global sensitivity analysis method developed by Sobol [46] to estimate the magnitude of the impact that variance in input parameters has on various model outputs.

Sets of input parameters were sampled from defined bounds using Saltelli’s scheme [47] and for each set the outputs were calculated as the means of 15 runs of the stochastic model. Parameter sampling and subsequent analysis was done using the Python library SALib version 1.4.7 [48], using the R package reticulate version 1.34.0 [49] to interface with R in order to run the model. We used total order Sobol indices as a measure of model sensitivity. These indices indicate what amount of variance in a designated model output is caused by variance in the various parameters in the analysis, taking into account interactions between the parameters [50].

We conducted four analyses—two concerning epidemiological and behavioural parameters and two concerning parameters associated with public health interventions. All parameters not included in a particular analysis were fixed at their calibrated values unless explicitly mentioned otherwise. This means that the analyses about epidemiological parameters involve transmission dynamics under contact tracing and pre-exposure vaccinations, and the analyses about intervention parameters assume transmission dynamics similar to the 2022 outbreaks. Unlike during the calibration process, here, we distinguished between the vaccination efficacy of the smallpox vaccination and the efficacy of the vaccine used for pre- and post-exposure vaccinations during the outbreak. This was done to make the analysis generalisable to situations where a different vaccine than the smallpox vaccine may be used as part of non-pharmaceutical interventions.

For all four analyses, we investigated the impact of the included parameters on the cumulative number of infections after 50, 100, and 365 days in, kindergarten-going children, care home residents, and the MSM subpopulation.

#### Analysis 1.1: Epidemiological and behavioural parameters, transmission similar to 2022 outbreaks

The first analysis contained three epidemiological and behavioural parameters that we identified as potentially highly impactful on mpox transmission dynamics during the 2022 outbreaks and for which there exist gaps in empirical knowledge. These are shown in Table 2, along with the ranges explored in the sensitivity analysis.

**Table 2:** Parameters included in sensitivity analysis 1.1, along with the ranges within which they were varied

Parameter	Range
Probability of transmission per sexual contact	[0.2, 0.7]
Ratio of sexual contacts of core MSM group to rest of MSM population	[10, 30]
Probability that previous smallpox vaccination confers immunity	[0.3, 1]

#### Analysis 1.2: Epidemiological and behavioural parameters, wider transmission

For this analysis, we included all the parameters from analysis 1.1, and also included the probability of transmission per non-sexual contact as a varying parameter (varied from 0.0 to 0.1), since theoretically mpox transmission is not limited to just sexual contacts.

#### Analysis 2.1: Intervention parameters, contact tracing, pre- and post-exposure vaccinations

In the first of two global sensitivity analyses about the impact of intervention parameters, we included two vaccination parameters—number of daily pre-exposure vaccinations offered and efficacy of the vaccine—and two tracing parameters, namely the base probability that a contact would be traced and the delay between a case being detected and their contacts being traced. The ranges explored for each of these parameters are given in Table 3.

**Table 3:** Parameters included in sensitivity analysis 2.1, along with the ranges within which they were varied

Parameter	Range
Mean number of daily pre-exposure vaccinations (integer)	[50, 250]
Probability of vaccine conferring immunity	[0.5, 1.0]
Base probability that a contact of a detected case will be traced	[0.1, 1.0]
Delay between case being detected and their contacts being traced (days) (integer)	[0, 10]

#### Analysis 2.2: Intervention parameters, contact tracing and post-exposure vaccinations

To investigate important intervention parameters in scenarios with low vaccine availability, we set the number of pre-exposure vaccinations to zero and ran a sensitivity analysis consisting of the tracing parameters included in analysis 2.1 and efficacy of (post-exposure) vaccinations.

## Results

### Calibration

The results of the model calibration process, including fitted parameter values, post-fitting checks, and trace plots for the pMCMC, are given in Supplementary File 2.

### Analysis of epidemiological and behavioural parameters

#### Analysis 1.1

We found that in our analysis of scenarios similar to the 2022 outbreaks (i.e. a very low transmission probability on non-sexual contact, contact tracing in place, and pre-and post-exposure vaccinations offered), variance in all included parameters contributed moderately to greatly to variance in the model outputs, as indicated by the total order Sobol indices in Table 4. The transmission probability per sexual contact was the dominant contributor to model dynamics throughout, with Sobol indices ranging from 0.61 to 0.76 across all outputs considered. Variance in the probability of a previous smallpox vaccination granting immunity contributed moderately to variance in the outputs and increased slightly over time (indicated by the Sobol indices for cumulative infections after 50 days being smaller than the Sobol indices for cumulative infections after 100 or 365 days). Variance in the ratio of sexual contacts of the core group to the non-core MSM group also contributed moderately to variance in the considered outputs, but this contribution decreased over time.

**Table 4:** Total order Sobol indices for the sensitivity analyses regarding analyses 1.1 and 1.2. The outputs considered are cumulative infections after 50, 100, and 365 days of simulation in the MSM community (MSM), kindergarten-going children (KG), and care home residents (Care)

*Total order Sobol indices for cumulative infection after:*	*50 days*			*100 days*			*365 days*		
	*MSM*	*KG*	*Care*	*MSM*	*KG*	*Care*	*MSM*	*KG*	*Care*
***Analysis 1.1.**** Epidemiological and behavioural parameters, transmission similar to 2022 outbreaks*									
*Probability of transmission on sexual contact*	0.62	0.68	*0.64*	*0.61*	*0.66*	*0.57*	*0.61*	*0.62*	*0.25*
*Probability of immunity given smallpox vaccination*	*0.28*	*0.15*	*0.32*	*0.33*	*0.27*	*0.47*	*0.33*	*0.42*	*0.76*
*Ratio of sexual contacts of core group to non-core group*	*0.38*	*0.40*	*0.38*	*0.27*	*0.30*	*0.27*	*0.25*	*0.10*	*0.09*
***Analysis 1.2.**** Epidemiological and behavioural parameters, wider transmission*									
*Probability of transmission on non-sexual contact*	0.00*	*0.68*	*0.73*	*0.01*	*0.81*	*0.84*	*0.62*	*0.99*	*0.94*
*Probability of transmission on sexual contact*	*0.61*	*0.39*	*0.31*	*0.60*	*0.33*	*0.12*	*0.22*	0.00*	0.00*
*Probability of immunity given smallpox vaccination*	*0.28*	*0.08*	*0.23*	*0.33*	*0.13*	*0.20*	*0.20*	0.00*	*0.08*
*Ratio of sexual contacts of core group to non-core group*	*0.40*	*0.19*	*0.19*	*0.26*	*0.13*	*0.05*	*0.04*	0.00*	0.00*

^*^ < 0.005, rounded to 0.00

#### Analysis 1.2

**Fig. 1:**
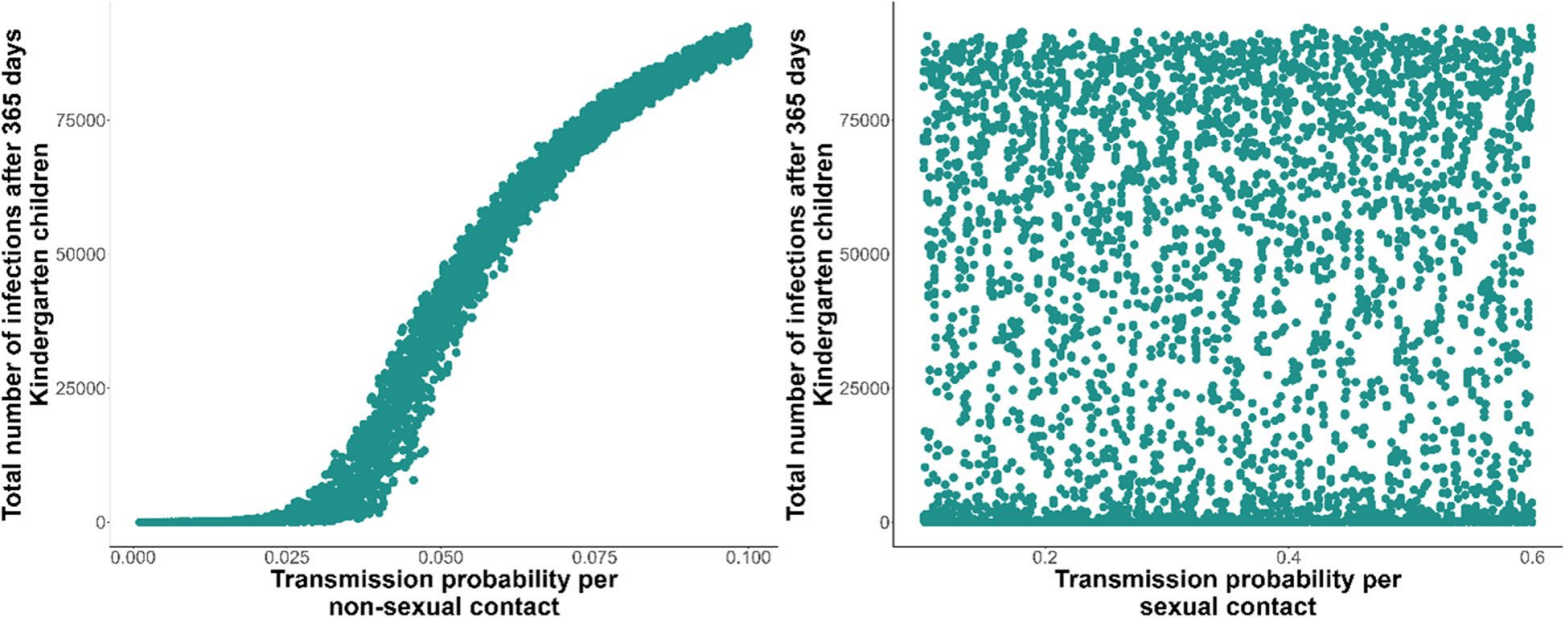
Cumulative infections in kindergarten-going children plotted against transmission probabilities. The clear vertical trend in the plot against transmission per non-sexual contact (left) indicates that cumulative infections in kindergarten-going children are very sensitive to this parameter, whereas the horizontal strata in the plot against transmission per sexual contact (right) on the right show that variance in this parameter is not a major contributor to variance in the number of cumulative infections in kindergarten-going children

When the transmission probability per non-sexual contact was also varied in the sensitivity analysis, we found that it was the dominant contributor to variance in all model outcomes except cumulative infection after 50 and 100 days in the MSM subpopulation. Variance in the transmission probability per sexual contact contributed moderately to variance in infections in the MSM subpopulation throughout and to a lesser extent cumulative infections after 50 and 100 days in care home residents and kindergarten-going children. However, it had close to no contribution to long-term dynamics—as indicated by cumulative infections after 365 days—in care home residents and kindergarten-going children. Figure 1 provides a visual demonstration of the differing sensitivity of cumulative infections after 365 days in kindergarten-going children to variance in the probabilities of transmission per non-sexual and sexual contact respectively.

The implication that, in this scenario, long-term mpox dynamics are mostly driven by non-sexual contacts is further strengthened by the Sobol indices for the ratio of sexual contacts of the core group to the no-core group; cumulative infections after 50 and 100 days in all three considered subpopulations are somewhat sensitive to this ratio, but it contributes very little to variance in the number of cumulative infections after 365 days in any considered subpopulation. Finally, infection numbers in the MSM subpopulation and care home residents were somewhat sensitive to the probability of immunity via previous smallpox vaccination, whereas infection numbers in kindergarten-going children were less sensitive to this parameter.

All Sobol indices from this analysis are displayed in Table 4.

### Analysis of intervention parameters

Since the sensitivity analyses of intervention parameters were conducted with epidemiological parameters reflecting the 2022 outbreaks, the probability of transmission per non-sexual contact was very low. This meant that the number of (cumulative) infections in kindergarten-going children and care home residents was small, especially after only 50 days of simulation. This makes interpretation of the Sobol indices for these outcomes difficult, and we do not discuss them below but have chosen to include them in the results table (Table 5) for the sake of completeness.

**Table 5:** Total order Sobol indices for the sensitivity analyses regarding analyses 2.1 and 2.2. The outputs considered are cumulative infections after 50, 100, and 365 days of simulation in the MSM community (MSM), kindergarten-going children (KG), and care home residents (Care)

*Total order Sobol indices for cumulative infection after:*	*50 days*			*100 days*			*365 days*		
	*MSM*	*KG*^a^	*Care*^a^	*MSM*	*KG*	*Care*	*MSM*	*KG*	*Care*
***Analysis 2.1.**** Intervention parameters, contact tracing, pre- and post-exposure vaccinations*									
*Delay in tracing*	0.04	0.21	*0.32*	*0.02*	*0.08*	*0.11*	*0.02*	*0.24*	*0.72*
*Base probability of being traced*	*0.02*	*0.17*	*0.39*	*0.02*	*0.08*	*0.13*	*0.03*	*0.49*	*0.24*
*Probability of vaccine immunising*	*0.18*	*0.27*	*0.38*	*0.29*	*0.20*	*0.18*	*0.34*	*0.15*	*0.06*
*Daily (mean) pre-exposure vaccinations*	*0.78*	*0.72*	*0.70*	*0.67*	*0.68*	*0.69*	*0.63*	*0.18*	*0.01*
***Analysis 2.2.**** Intervention parameters, contact tracing and post-exposure vaccinations*									
*Delay in tracing*	*0.74*	*0.76*	*0.80*	*0.67*	*0.58*	*0.61*	*0.65*	*0.34*	*0.24*
*Base probability of being traced*	*0.42*	*0.53*	*0.68*	*0.41*	*0.50*	*0.48*	*0.42*	*0.66*	*0.75*
*Probability of vaccine immunising*	*0.04*	*0.28*	*0.42*	*0.02*	*0.01*	*0.02*	*0.03*	*0.01*	*0.03*

^a^There was very little variance in absolute terms in these outputs, due to low non-sexual transmission. Sobol indices for these terms should thus be interpreted with caution

#### Analysis 2.1

We found that, when pre-exposure vaccinations were implemented in addition to contact tracing and post-exposure vaccinations, variance in the parameters governing vaccination, i.e. the mean daily number of pre-exposure vaccinations offered and the efficacy of the vaccine, contributed greatly to variance in cumulative infections in the MSM community (as shown by the Sobol indices in Table 5). This dwarfed the contribution of variance in the tracing delay and base tracing probability. In contrast, variance in the tracing parameters contributed more than variance in vaccination parameters to cumulative infection variance in kindergarten-going children and care home residents, especially in cumulative infection after 365 days.

#### Analysis 2.2

**Fig. 2:**
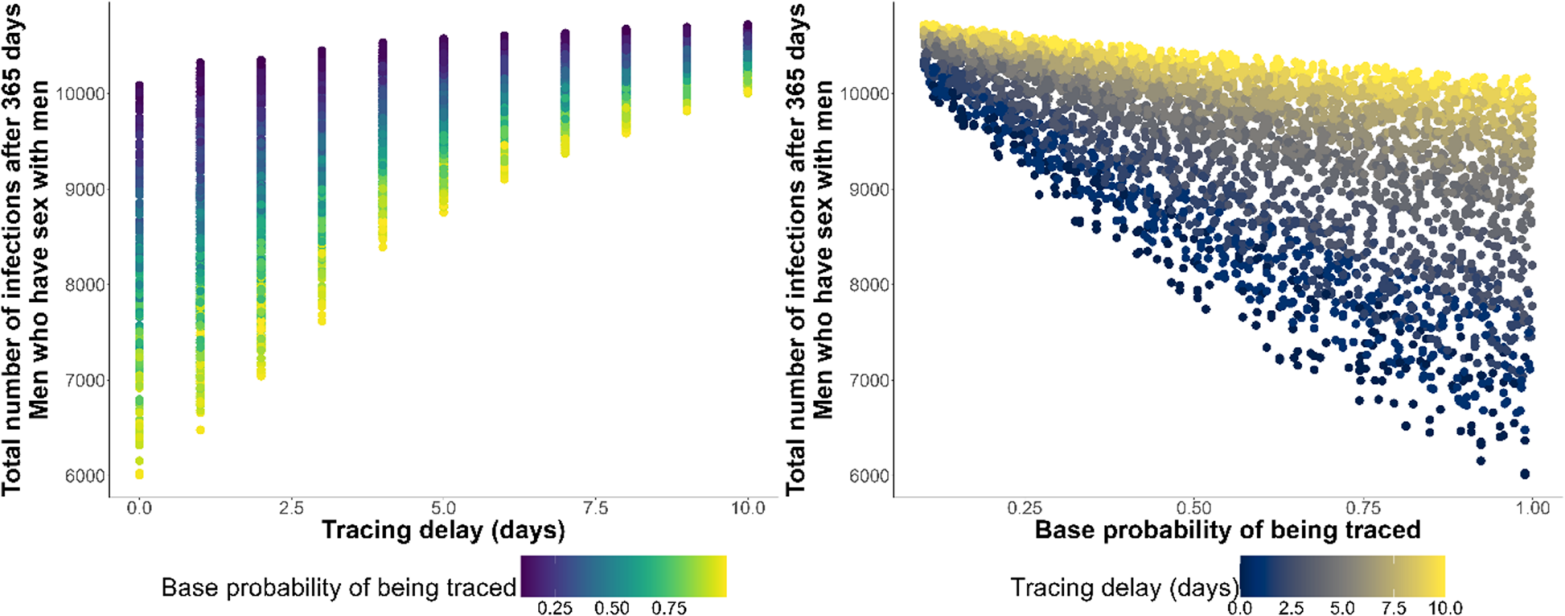
Cumulative infections in the MSM community plotted against contact tracing parameters. The plot shows cumulative infections in the MSM community plotted against (left) delay in tracing with colour indicating base probability of being traced and (right) base probability of being traced with colour indicating delay in tracing in analysis 2.2. The clear vertical trends indicate the sensitivity of the infection dynamics in the MSM community to both tracing parameters of cumulative infections in the MSM community to both these parameters in analysis 2.2

When pre-exposure vaccinations were not in place, we found that all considered model outputs were very sensitive to both the delay in contact tracing and the base probability that a contact would be traced. This can be seen in the Sobol indices in Table 5 and the scatter plots in Fig. 2. Variance in the efficacy of (post-exposure) vaccinations contributed far less to output variance than either tracing parameter.

## Discussion

The model we have presented here allows for the simulation of an epidemic of mpox or a similar disease in different high-contact settings and for the realistic implementation and analysis of non-pharmaceutical interventions to contain the epidemic. The model is tailored not towards prediction modelling or exact forecasts, but towards comparing scenarios and non-pharmaceutical interventions, and identifying parameters that have a large impact on the course of an mpox outbreak.

Estimates of the secondary attack rate—an approximation for the per-contact transmission probability—from previous outbreaks [14] do not distinguish between sexual and non-sexual contacts. Our findings showed that the infection dynamics of mpox are very sensitive to the transmission probability per non-sexual contact. Variance in the transmission probability per sexual contact contributed somewhat to variance in the short-term dynamics, but in the long run, its effect was dwarfed by that of transmission through non-sexual contacts, indicating that in these scenarios the epidemic was triggered by sexual contacts but sustained by non-sexual contacts. This could simply be due to the fact that the majority of physical contacts are not sexual. However, if the transmission probability per non-sexual contact was very low, as we believe it to be—and as the calibration results suggest—in the outbreaks of 2022, the course of the epidemic was very sensitive to the transmission probability per sexual contact throughout, and the epidemic could only be sustained by sexual contacts. Therefore, a precise understanding and quantification of both these parameters separately is necessary to support modelling efforts for future outbreaks. However, precise estimation of transmission probabilities is always challenging, and estimation of the transmission probability per sexual contact in particular is often hindered by the intimate—and sometimes anonymous—nature of sexual contacts. The same is true for trying to quantify the higher level of sexual activity of the core group, which is the other sexual-associated parameter we investigated and found dynamics to be sensitive to.

It is to be expected that the immunity provided by the smallpox vaccine will have more of an impact on infection dynamics in the more elderly age groups, i.e., care home residents and to some extent the MSM population than the younger ones such as kindergarten-going children, and this is what we saw in our analyses. However, we found that even under these assumptions the infection dynamics in children and the MSM community—which was skewed towards the younger age groups—were moderately sensitive to the level of such immunity. Despite the limitations of our simplistic model and assumptions of smallpox vaccinations and associated mpox immunity, it is clear that this immunity plays a crucial role in the dynamics of an mpox outbreak. Therefore, attention needs to be paid to how it is modelled in infectious disease models for mpox, and studies that can establish the level of pre-existing immunity to mpox in the population due to the smallpox vaccination will be very useful to enable correct parametrisation of the same.

When non-pharmaceutical interventions consisted of targeted pre-exposure vaccinations in addition to contact tracing and post-exposure vaccinations, we found that the number of pre-exposure vaccinations and vaccine efficacy contributed the most to variance in cumulative infections in the MSM community, but the tracing parameters contributed more to variance in cumulative infections in kindergarten-going children and care home residents. This is likely due to the facts that pre-exposure vaccinations were only administered in the MSM ‘core group’ and infections were also initialised in this group, spreading to kindergarten-going children and care home residents only through secondary contacts. This could imply that, in scenarios similar to the recent outbreaks, effective contact tracing could help control the spread of mpox, but improving vaccine efficacy and distribution would help more towards entirely preventing an epidemic. However, it should be noted that vaccine development and procurement is challenging and expensive, and improvements may be more difficult to achieve.

Furthermore, in the absence of pre-exposure vaccinations, we found that transmission dynamics even in the MSM community were sensitive to both the base probability of being traced, which can loosely be interpreted as tracing accuracy and the lag between an infectious person being detected and their contacts being traced. We also found that these parameters were interdependent, with accurate but slow tracing, as well as fast but inaccurate tracing not being very effective at reducing infection numbers. Since tracing accuracy and tracing speed are antagonistic to an extent, our results suggest that a balance must be found between the two for effective contact tracing.

We found variance in vaccine efficacy to have a relatively smaller effect on the infection dynamics when the only way to administer vaccinations was through post-exposure vaccination. This is likely due to the fact that post-exposure vaccination is inherently dependent on contact tracing, and therefore the effect of variance in the tracing parameters dominates over the effect of vaccine efficacy. This does not imply that post-exposure vaccination is not an effective control strategy. Indeed, there is modelling-based evidence that it can be very effective at containing smallpox outbreaks [51], and there is no reason to believe that this should be different with mpox.

One large limitation of this study is the contact matrix framework we have used (and which is required by the compartmental model approach). This implicitly assumes some level of homogeneous population mixing, which might not be fully appropriate for the types of contacts considered in the model. The same approach also makes it difficult to model super-spreading events, and there is evidence that these played a large role in the recent outbreaks [52]. However, we have tried to mitigate this by explicitly modelling a core group of individuals with an increased frequency of sexual contacts, and the calibration and validation results in Supplementary File 2 indicate that our approach can successfully model mpox dynamics in the general population. Furthermore, the parameters used to derive the contact matrices were often based on generalisations of studies and literature from different settings and countries, and the validity of our contact matrices relies on the assumption that these generalisations are valid. As an example for how this may affect the study, a very low transmission probability is not the only explanation for low observed incidence in children and non-MSM communities during the 2022 outbreaks; since the outbreak likely started in the MSM community, it could simply be that the number of non-sexual physical contacts between the MSM community, especially the core high activity group, and children and other non-MSM communities is much lower than our derived contact matrices imply. Another limitation is that, although some reinfections have been reported [53], our model does not account for the possibility of reinfection with mpox. However, the number of reinfections is small, and we do not believe that it has a large impact on mpox outbreak dynamics. Neither does our model account for other transmission routes such as from contaminated objects and injuries in health care settings, tattoo parlours, and the like, but similarly we do not believe that these routes contribute significantly to the overall disease dynamics on a population level. Finally, we have generalised the sensitivity analysis of our model to mpox models and dynamics in general. However, as with any modelling-based study, it is possible that our results are artefacts of the specific model we chose and have limited applicability otherwise.

Despite these limitations, we believe that our model encapsulates the main features of mpox outbreak dynamics and associated non-pharmaceutical interventions and provides a reasonable alternative to more complex agent-based and network models.

## Conclusions

Our study suggests that further research and precise quantification of parameters such as the SAR and the level of immunity to mpox provided by the smallpox vaccination, through region and population-specific epidemiological and behavioural studies, is necessary to enable infectious disease modelling efforts—irrespective of the type of model used—and be better prepared for future outbreaks. Our study also suggests that improving vaccine efficacy and distribution of pre-exposure vaccinations would be an effective way of containing an mpox outbreak and that the success of contact tracing and post-exposure vaccinations depends on the speed and accuracy of tracing equally.

### Supplementary Information

Supplementary Material 1: Formal description of mathematical model, Fig. S1.1, Tables S1.1–S1.4. Fig. S1.1: Model schematic. Table S1.1: Contact and infection events in model. Table S1.2: Transition events in model. Table S1.3: Events related to control strategies. Table S1.4: Sources for demography parameters used.

Supplementary Material 2: Calibration and Validation of Model, Figs. S2.1–S2.3, Tables S2.1–S2.4. Fig. S2.1: Trace and density plots for Markov Chain Monte Carlo run. Fig. S2.2: Fitted model trajectories plotted against data used for fit. Fig. S2.3: Fitted model trajectories plotted against data not used for fit. Table S2.1: Bounds on parameters to be calibrated. Table S2.2: Initial values and proposal matrix variances for Markov Chain Monte Carlo run. Table S2.3: Labels for parameters used in figures. Table S2.4: Mean post-calibration values of parameters as used in the model.

## Data Availability

All the code and data used for this manuscript are available at https://zivgitlab.uni-muenster.de/chaturve/mpox.

## Abbreviations

KG: Kindergarten-going children
MSM: Men who have sex with men
