# Supplementary material for: Estimating the relative importance of epidemiological and behavioural parameters for epidemic mpox transmission: a modelling study
Source: BMC Med. 2024 Jul 18;22:297. doi: 10.1186/s12916-024-03515-8 (PMC11256368; doi:10.1186/s12916-024-03515-8)
Supplement: Supplementary file 1 — Supplementary Material 1: Formal description of mathematical model, Fig. S1.1, Tables S1.1–S1.4. Fig. S1.1: Model schematic. Table S1.1: Contact and infection events in model. Table S1.2: Transition events in model. Table S1.3: Events related to control strategies. Table S1.4: Sources for demography parameters used. [file 12916_2024_3515_MOESM1_ESM.pdf]

# Mpox Model Definition

## 1 Structure

The model's core is a compartmental Susceptible, Exposed (i.e. infected but pre-infectious), Infectious, Recovered (SEIR) model, modified to accommodate underdetection of infection, isolation upon detection of infection, and quarantining of traced contacts. To this end, the Infectious compartment is split into 'Detectable' and 'Undetectable' compartments, and there are additional compartments for 'Susceptible in quarantine', 'Exposed in quarantine', and '(Detected) infectious in isolation'. We do not explicitly consider deaths due to mpox in our analyses, and therefore combine recovery and death into the Recovered/Deceased compartments. If desired, explicit modelling of deaths can be achieved by splitting these compartments. A slightly simplified model schematic is presented in Figure S1.1.

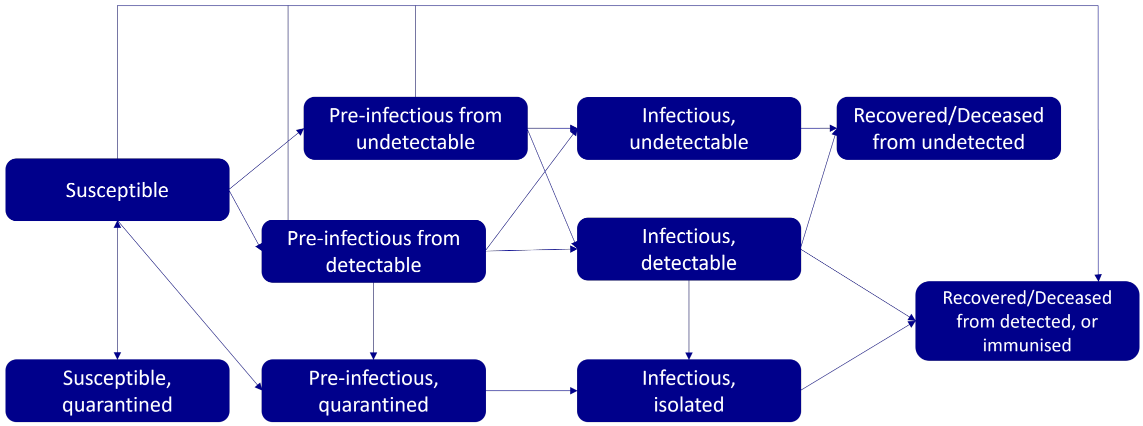


Figure S1.1: Simplified schematic for model. Not shown are subcompartments for each compartment that track 'time since event', and subcompartments that track vaccination status in relevant compartments

# 2 Compartments and subcompartments

Below are the definitions, notation, and other detail of all the compartments and subcompartments included in the model, sorted by infection status. The general schema for notation is as follows: Each compartment has a global time index $t$, that tracks time since start of simulation, and some compartments have secondary time indices denoted by $\tau$, that track 'time since event', for events depending on the exact compartment. Time indices are always subscripts. Furthermore, for some compartments it is important to track vaccination status (especially if the vaccination was unsuccessful, since unsuccessfully vaccinated people will not see a change in infection status but will not be offered the vaccine again). A superscript $V$ denotes the vaccinated subcompartment of the relevant compartment, whereas a superscript $N$ denotes the non-vaccinated subcompartment. A superscript $Q$ is used to signify compartments that deal with quarantine or isolation, and superscripts $D$ and $U$ differentiate between detectable and undetectable infection in some way.

## Susceptible compartments

### Susceptible, not in quarantine ($S$)

This compartment tracks the number of individuals who are susceptible to the disease, and are not quarantining and can thus be exposed to the disease. It is denoted by $S^V_{t,\tau}$ and $S^N_{t,\tau}$, signifying the number of such vaccinated and unvaccinated individuals respectively. The secondary time index $\tau$ denotes the number of days since last contact with a detectable infectious person, which is important for contact tracing, with $\tau = 0$ being the individuals who have never had contact with a detectable infectious individual or whose last such contact was more than 21 days (maximum duration of infectious period and hence maximum duration of contact tracing) ago.

### Susceptible in quarantine ($S^Q$)

This compartment tracks the number of individuals who are susceptible to the disease but are currently in quarantine due to contact tracing. This is denoted by $S^Q_{t,\tau}$, and the secondary time index $\tau$ tracks the number of days spent in quarantine. Tracking vaccination status is not needed here since everyone who is identified by contact tracing is also vaccinated.

## Exposed/pre-infectious compartments

### Pre-infectious from detectable, not in quarantine ($E^D$)

This subcompartment tracks the number of people who are infected but not yet infectious, were infected by a person with detectable infection and therefore can be identified by contact tracing, but are not in quarantine yet. Vaccination status is tracked for this compartment, and the subcompartments for vaccinated and non-vaccinated are denoted $E_{t,\tau}^{D,V}$ and $E_{t,\tau}^{D,N}$ respectively. The secondary time index $\tau$ tracks time since infection, which also doubles as time since contact with detectable infectious person for purposes of contact tracing.

### Pre-infectious from detectable, in quarantine ($E^Q$)

This subcompartment tracks the number of people who are infected but not yet infectious, and are in quarantine due to contact tracing. This is denoted by $E_{t,\tau}^{Q}$, with $\tau$ tracking time since infection, and similarly to susceptible quarantine, tracking of vaccination status is not necessary here since contact tracing goes hand-in-hand with ring vaccination.

### Pre-infectious from undetectable ($E^U$)

This compartment keeps track of people who are infected but not yet infectious, and were infected by someone with undetectable infection. Thus they are not identifiable by contact tracing. To simplify the model, we assume that any contacts people in this compartment have with detectable infectious individuals cannot lead to contact tracing. Vaccination status is tracked in this compartment, giving rise to the subcompartments $E_{t,\tau}^{U,V}$ and $E_{t,\tau}^{U,N}$. As in the other exposed compartments, $\tau$ tracks time since infection. The model is run using a tau-leaping scheme, where the number of events that happen each day is determined from certain probability distributions, as described below.

## Infectious compartments

### Infectious, detectable, not in isolation ($I^D$)

This compartment tracks the number of people that are infectious, can be detected as such (but may recover before this happens), but have not yet

been detected and are thus not in isolation and can spread the disease. The secondary time index tracks time since turning infectious. Vaccination status is tracked since we assume that people in this compartment can be offered vaccinations until they are detected to be infectious (but vaccination in this compartment does not have an effect on infection status). Thus we have the subcompartments $I_{t,\tau}^{D,V}$ and $I_{t,\tau}^{D,N}$.

### Infectious, in isolation ($I^Q$)

This compartment tracks the number of people who are infectious but are in isolation due to either being detected as infectious, or having been quarantined when they were pre-infectious and then turning infectious. Vaccination status is not tracked here since people with detected infection are not offered the vaccination. This compartment is denoted by $I_{t,\tau}^{Q}$, with $\tau$ tracking time since turning infectious.

### Infectious, undetectable ($I^U$)

This compartment tracks the number of people that are infectious, but whose infection is undetectable. People in this compartment will not be put into isolation, and are still offered vaccinations (but vaccination in this compartment does not have an effect on infection status). Thus we have subcompartments $I_{t,\tau}^{U,V}$ and $I_{t,\tau}^{U,N}$, with $\tau$ denoting time since turning infectious.

## Recovered/Reomved/Immune compartments

### Recovered from detected infection, or immune due to vaccination ($R^D$)

This compartment includes people who die due to a detected infection, have recovered from a detected infection (we make the simplifying assumption that people with detectable infection will be detected at some point, be it before or after recovery), or have been successfully immunised by vaccination. We do not need to track vaccination status here, nor do we need a secondary time index. Hence, this compartment is denoted $R_t^D$.

**Recovered/Removed from undetectable infection ($R^U$)**

This compartment tracks the number of people who have recovered or died from an undetectable infection. No secondary time index is needed for this compartment, but such people can be offered vaccinations so we track vaccination status. Thus we have the subcompartments $R_t^{U,V}$ and $R_t^{U,N}$.

# 3 Dynamics

Note: For clarity of notation, we introduce the dynamics without talking about metapopulations or age stratification, treating each class/compartment as a scalar. In reality, all the classes/quantities are vectors, with one quantity per population stratum, and mean contacts are encoded in contact matrices and handled accordingly.

The model is simulated using a tau leaping approach, where a number of events is drawn from an appropriate distribution at each time step, and compartments are updated based on these events. In a slight abuse of notation when describing events, we will use the labels of each compartment defined above to indicate both the compartment itself, and the number of people in the compartment.

The type, label, and density distribution of all events are described below. Firstly, we determine the number of relevant contact and infection events as in Table S1.1 (with the notation convention that $I_t^x = \sum_\tau \sum_{y \in \{V,N\}} I_{t,\tau}^{x,y}$, for $x \in \{D, U\}$).

| **Event** | **Label** | **Distribution** |
| --- | --- | --- |
| Non-sexual contacts between $S^y_{t,\tau}$ and $I^D_t$, for $y \in \{V, N\}$ | $C^{D,y}_{t,\tau}$ | $\text{Pois}\left(\frac{cS^y_{t,\tau}I^D_t}{N_p}\right)$ |
| Non-sexual contacts between $S^y_{t,\tau}$ and $I^U_t$ , for $y \in \{V, N\}$ | $C^{U,y}_{t,\tau}$ | $\text{Pois}\left(\frac{c\left(S^y_{t,\tau} - C^{D,y}_{t,\tau}\right)I^U_t}{N_p}\right)$ |
| Sexual contacts between $S^y_{t,\tau}$ and $I^D_t$, for $y \in \{V, N\}$ | $C^{s,D,y}_{t,\tau}$ | $\text{Pois}\left(\frac{c^s\left(S^y_{t,\tau} - C^{D,y}_{t,\tau} - C^{U,y}_{t,\tau}\right)I^D_t}{N_p}\right)$ |
| Sexual contacts between $S_{t,\tau}$ and $I^U_t$, for $y \in \{V, N\}$ | $C^{s,U,y}_{t,\tau}$ | $\text{Pois}\left(\frac{c^s\left(S^y_{t,\tau} - C^{D,y}_{t,\tau} - C^{U,y}_{t,\tau} - C^{s,D,y}_{t,\tau}\right)I^U_t}{N_p}\right)$ |
| All contacts between $S^y_{t,\tau}$ and $I^x_t$ that lead to infection, for $x \in \{D, U\}$, $y \in \{V, N\}$ | $\iota^{x,y}_{t,\tau}$ | $\text{Bin}\left(C^{x,y}_{t,\tau},\ \beta\right)+\text{Bin}\left(C^{s,x,y}_{t,\tau},\ \beta^s\right)$ |

Table S1.1: Contact and infection events

$c$ is the mean number of non-sexual contacts per day, $c^s$ is the number of sexual contacts per day, $N_p$ is the population size, $\beta$ is the non-sexual secondary attack rate, and $\beta^s$ is the sexual secondary attack rate.

It is important to note that we have implicitly assumed above that there is no overlap between sexual and non-sexual contacts, or contacts with detectable and undetectable infectious individuals.

Transition events from the Exposed and Infectious compartments are determined as in Table S1.2. For brevity, we use the notation $E^{x,y}$ with $x \in \{D, U, Q\}, y \in \{V, N\}$, although vaccination status is not tracked in

$E^Q$. Therefore, it shold be understood that, for cases where $x = Q$, the vaccination superscript $y$ is not applied. The same holds for $I^Q$.

| **Event** | **Label** | **Distribution** |
|---|---|---|
| $E^{x,y}_{t,\tau}$ becoming infectious, for $x \in \{D, U, Q\}, y \in \{V, N\}$ | $\epsilon^{x,y}_{t,\tau}$ | $\text{Bin}\big(E^{x,y}_{t,\tau},\ \sigma_\tau\big)$ |
| Of the individuals not in quarantine becoming infectious ($\epsilon^{x,y}_{t,\tau}$), the number that will be detectable, for $x \in \{D, U\}, y \in \{V, N\}$ | $\epsilon^{*x,y}_{t,\tau}$ | $\text{Bin}\big(\epsilon^{x,y}_{t,\tau},\ d\big)$ |
| $I^{x,y}_{t,\tau_I}$ recovering or dying, for $x \in \{D, U, Q\}, y \in \{V, N\}$ | $G^{x,y}_{t,\tau_I}$ | $\text{Bin}\big(I^{x}_{t,\tau_I},\ g_{\tau_I}\big)$ |
| $I^{D,y}_{t,\tau_I}$ being detected, for $y \in \{V, N\}$ | $\Delta^{y}_{t,\tau_I}$ | $\text{Bin}\Big(I^{D,y}_{t,\tau_I},\ \delta_{\tau_I}\Big)$ |

Table S1.2: Transition events concerning the Exposed and Infectious compartments

$\sigma_\tau$ denotes the probability that an Exposed individual on day $\tau$ of their incubation period will become infectious on the next day. $d$ is the probability that an infection will be detectable, $g_{\tau_I}$ is the probability that an infectious person on day $\tau_I$ of their infectious period will recover on that day, and $\delta_{\tau_I}$ is the probability that a detectable but not yet detected infectious person on day $\tau_I$ of their infectious period will be detected on that day. Details about these probabilities will follow later. We make the assumption that Exposed quarantining individuals will remain in isolation until they recover regardless of whether their infectious period would be detectable or not.
Finally, we determine events related to control strategies as in Table S1.3. Pre-exposure vaccinations are defined generally as happening in compartments $X^N_{t,\tau}$, for relevant $X$. Since the recovered compartments do not have a secondary time index, the subscript $\tau$ is not applied here when $X = R^U$.

| **Event** | **Label** | **Distribution** |
|---|---|---|
| Number of $S^y_{t,\tau}$ traced, for $y \in \{V, N\}$ | $T^{S,y}_{t,\tau}$ | $\text{Bin}\big(S^y_{t,\tau},\ \theta_{t,\tau}\big)$ |
| Successful vaccination of traced susceptible contacts | $V^S_{t,\tau}$ | $\text{Bin}\Big(T^{S,N}_{t,\tau},\ v\mathbb{1}_{\tau\leq 14}\Big)$ |
| Number of $E^{D,y}_{t,\tau}$ traced, for $y \in \{V, N\}$ | $T^{E,y}_{t,\tau}$ | $\text{Bin}\Big(E^{D,y}_{t,\tau},\ \theta_{t,\tau}\Big)$ |
| Successful vaccination of traced exposed contacts | $V^E_{t,\tau}$ | $\text{Bin}\Big(T^{E,N}_{t,\tau},\ v\mathbb{1}_{\tau\leq 4}\Big)$ |
| Pre-exposure vaccination in compartment $X^N_{t,\tau}$, for $X \in \{S, E^U, E^D, I^U, I^D, R^U\}$ | $P^X_{t,\tau}$ | $\text{Pois}\left(\dfrac{nX^N_{t,\tau}}{N^v_t}\right)$ |
| Successful pre-exposure vaccination of susceptibles | $V^{*S}_{t,\tau}$ | $\text{Bin}\big(P^S_{t,\tau},\ v\big)$ |
| Successful pre-exposure vaccination of exposed, $x \in D, U$ | $V^{*E^x}_{t,\tau}$ | $\text{Bin}\big(P^{E^x}_{t,\tau},\ v\mathbb{1}_{\tau\leq 4}\big)$ |

Table S1.3: Events related to control strategies

$\theta_{t,\tau}$ is the probability of being traced on day $t$ given that the last contact with a detectable infectious person was $\tau$ days ago (details about how this is calculated are given later), $v$ is the probability that a traced person will be successfully vaccinated, $N^v_t$ is the total number of people eligible for pre-exposure vaccination at global time $t$, and $n$ is the number of pre-exposure vaccinations available daily. The current guidelines from the Robert Koch Institute [20] suggest that, during ring vaccination, only those people should be vaccinated whose exposure was less than 14 days ago, therefore the vaccination probability for traced susceptible contacts is set to 0 by the indicator function if $\tau > 14$. Furthermore, we assume in line with guidelines [21] that Exposed individuals can be successfully immunised by vaccination in the first 4 days of their pre-infectious period but not after. Therefore, we have another indicator function that sets vaccination probabilities in the exposed

compartments to 0 after day 4 of their pre-infectious period.

Then, adopting the notation $X_{t,-1}$ to denote the maximum possible $\tau$ for any class labelled $X_{t,\tau}$, we have the following rules to update the various compartments:

$$\begin{aligned} S^N_{t+1,0} =& S^N_{t,0} - C^{D,N}_{t,0} - C^{s,D,N}_{t,0} - \iota^{U,N}_{t,0} \\ &+ S^N_{t,-1} - C^{D,N}_{t,-1} - C^{s,D,N}_{t,-1} - T^{S,N}_{t,-1} - \iota^{U,N}_{t,-1} \\ &- P^S_{t,-1} - P^S_{t,0} \end{aligned} \tag{1}$$

$$\begin{aligned} S^V_{t+1,0} =& S^V_{t,0} - C^{D,V}_{t,0} - C^{s,D,V}_{t,0} - \iota^{U,V}_{t,0} \\ &+ S^V_{t,-1} - C^{D,V}_{t,-1} - C^{s,D,V}_{t,-1} - T^{S,V}_{t,-1} - \iota^{U,V}_{t,-1} \\ &+ S^Q_{t,-1} + P^S_{t,-1} + P^S_{t,0} \\ &- V^{*S}_{t,-1} - V^{*S}_{t,0} \end{aligned} \tag{2}$$

$$S^y_{t+1,1} = \sum_\tau C^{D,y}_{t,\tau} + \sum_\tau C^{s,D,y}_{t,\tau} - \sum_\tau \iota^{D,y}_{t,\tau}, \text{ for } y \in \{N, V\} \tag{3}$$

$$S^N_{t+1,\tau+1} = S^N_{t,\tau} - C^{D,N}_{t,\tau} - C^{s,D,N}_{t,\tau} - \iota^{U,N}_{t,\tau} - T^{S,N}_{t,\tau} - P^S_{t,\tau}, \text{ for } 1 \le \tau < 21 \tag{4}$$

$$\begin{aligned} S^V_{t+1,\tau+1} =& S^V_{t,\tau} - C^{D,V}_{t,\tau} - C^{s,D,V}_{t,\tau} - \iota^{U,V}_{t,\tau} - T^{S,V}_{t,\tau} + P^S_{t,\tau} - V^{*S}_{t,\tau} \\ &\text{for } 1 \le \tau < 21 \end{aligned} \tag{5}$$

$$S^Q_{t+1,1} = \sum_\tau \left( \sum_{y \in \{N,V\}} T^S_{t,\tau} - V^S_{t,\tau} \right) \tag{6}$$

$$S^Q_{t+1,\tau_Q+1} = S^Q_{t,\tau_Q}, \text{ for } 1 \le \tau_Q < 21 \tag{7}$$

$$E^{x,y}_{t+1,1} = \sum_\tau \iota^{x,y}_{t,\tau} \text{ for } x \in \{D, U\},\ y \in \{N, V\} \tag{8}$$

$$E^{D,N}_{t+1,\tau+1} = E^{D,N}_{t,\tau} - \epsilon^{D,N}_{t,\tau} - T^{E,N}_{t,\tau} - P^{E^D}_{t,\tau} \text{ for } 1 \leq \tau < 21 \tag{9}$$

$$E^{D,V}_{t+1,\tau+1} = E^{D,V}_{t,\tau} - \epsilon^{D,V}_{t,\tau} - T^{E,V}_{t,\tau} + P^{E^D}_{t,\tau} - V^{*E^D}_{t,\tau} \text{ for } 1 \leq \tau < 21 \tag{10}$$

$$E^{U,N}_{t+1,\tau+1} = E^{U,N}_{t,\tau} - \epsilon^{U,N}_{t,\tau} - P^{E^U}_{t,\tau} \text{ for } 1 \leq \tau < 21 \tag{11}$$

$$E^{U,V}_{t+1,\tau+1} = E^{U,V}_{t,\tau} - \epsilon^{U,V}_{t,\tau} + P^{E^U}_{t,\tau} - V^{*E^U}_{t,\tau} \text{ for } 1 \leq \tau < 21 \tag{12}$$

$$E^{Q}_{t+1,\tau+1} = E^{Q}_{t,\tau} - \epsilon^{Q}_{t,\tau} + \sum_{y \in \{N,V\}} T^{E,y}_{t,\tau} - V^{E}_{t,\tau} \text{ for } 1 \leq \tau < 21 \tag{13}$$

$$I^{D,y}_{t+1,1} = \sum_{x \in \{D,U\}} \sum_{\tau} \epsilon^{*x,y}_{t,\tau}, \text{ for } y \in \{N, V\} \tag{14}$$

$$I^{U,y}_{t+1,1} = \sum_{x \in \{D,U\}} \sum_{\tau} \left( \epsilon^{x,y}_{t,\tau} - \epsilon^{*x,y}_{t,\tau} \right), \text{ for } y \in \{N, V\} \tag{15}$$

$$I^{Q}_{t+1,1} = \sum_{\tau} \epsilon^{Q}_{t,\tau} \tag{16}$$

$$I^{D,N}_{t+1,\tau_I+1} = I^{D,N}_{t,\tau_I} - G^{D,N}_{t,\tau_I} - \Delta^{N}_{t,\tau_I} - P^{I^D}_{t,\tau} \text{ for } 1 \leq \tau_I < 21 \tag{17}$$

$$I^{D,V}_{t+1,\tau_I+1} = I^{D,V}_{t,\tau_I} - G^{D,V}_{t,\tau_I} - \Delta^{V}_{t,\tau_I} + P^{I^D}_{t,\tau} \text{ for } 1 \leq \tau_I < 21 \tag{18}$$

$$I^{U,N}_{t+1,\tau_I+1} = I^{U,N}_{t,\tau_I} - G^{U,N}_{t,\tau_I} - P^{I^U}_{t,\tau} \text{ for } 1 \leq \tau < 21 \tag{19}$$

$$I^{U,V}_{t+1,\tau_I+1} = I^{U,V}_{t,\tau_I} - G^{U,V}_{t,\tau_I} + P^{I^U}_{t,\tau} \text{ for } 1 \leq \tau < 21 \tag{20}$$

$$I^{Q}_{t+1,\tau_I+1} = I^{Q}_{t,\tau_I} - G^{Q}_{t,\tau_I} + \sum_{y \in \{N,V\}} \Delta_{t,\tau_I} \text{ for } 1 \leq \tau_I < 21 \tag{21}$$

$$
\begin{aligned}
R^{D}_{t+1} =& R^{D}_{t} + \sum_{\tau_I} \left( G^{Q}_{t,\tau_I} + \sum_{y \in \{N,V\}} G^{D,y}_{t,\tau_I} \right) + \sum_{X \in \{S,E\}} \sum_{\tau} V^{X}_{t,\tau} \\
&+ \sum_{X \in \{S,E^D,E^U\}} \sum_{\tau} V^{*X}_{t,\tau}
\end{aligned} \tag{22}
$$

$$
R^{U,N}_{t+1} = R^{U,N}_{t} + \sum_{\tau_I} G^{U,N}_{t,\tau_I} - P^{R^U}_{t} \tag{23}
$$

$$
R^{U,V}_{t+1} = R^{U,V}_{t} + \sum_{\tau_I} G^{U,V}_{t,\tau_I} + P^{R^U}_{t} \tag{24}
$$

There are some edge cases that need to be handled separately, occurring when two events happen at the same time. For example, someone in $S_{t,\tau}$ ($\tau > 0$) who gets contacted by a detectable infectious person and also traced (from their previous contact) on day $t$ must be put into $S^{Q}_{t+1,1}$ as opposed to $S_{t+1,1}$. We have omitted such edge-case handling from the equations above, but they are handled in the python script that runs the model.

# 4 Parameters

## Incubation Period

We assume, in line with existing evidence [22], that the incubation period follows a lognormal distribution, with parameters $\mu = 2.09$, $\sigma = 0.44$. We truncate this distribution to between 5 and 21 days and discretise it as follows:

$$
F_t(x) = \frac{F(x) - F(4)}{F(21) - F(4)} \tag{25}
$$

$$
\mathbb{P}(X = x) = F_t(x) - F_t(x-1) \;\forall x \in \{5, 21\} \tag{26}
$$

where $F$ is the cumulative distribution function (cdf) of the lognormal distribution, $F_t$ the cdf of the truncated lognormal distribution, and $\mathbb{P}$ the ensuing probability mass function (pmf) (where $X$ is the random variable encoding

the incubation period). This pmf is given in **summary.xlsx**.
The probability $\sigma_\tau$ used in the model equations above is the conditional probability that the incubation period will last $\tau$ days given that it has lasted longer than $\tau - 1$ days. This is obtained by the equation:

$$\sigma_\tau = \mathbb{P}(X = \tau | X \geq \tau) = \frac{\mathbb{P}(X = \tau)}{\sum_{i=\tau}^{21} \mathbb{P}(X = i)} \tag{27}$$

## Infectious Period

We assumed that the infectious period follows a gamma distribution, with parameters $\alpha = 5$, $\beta = 3$, truncated to between 10 and 21 days. The pmf given for the infectious period in **summary.xlsx** is the pmf of this discretised, truncated distribution, obtained exactly the same way as the pmf for the incubation period, described above. The probabilities $g_{\tau_I}$ used in the model equations are obtained from this pmf the same way as $\sigma_\tau$ above.

## Detection Probabilities

We assume that a proportion of the infectious individuals will be detectable ($I^D$), that if these individuals were to never recover, they would all be eventually detected, and that their day of detection would follow a truncated, discretised gamma distribution with parameters $\alpha = 3$, $\beta = 2$, truncated between 3 and 21 days. The pmf of this distribution is obtained from the cdf of the gamma distribution the same way as for the infectious period. Finally, the probabilities $\delta_{\tau_I}$ used in the model are obtained from this pmf, as described above.

## Probability of being traced

The probabilities $\theta_{t,\tau}$ used in the model represent the probability that a susceptible or exposed person who last had contact with a detectable infectious person $\tau$ days ago are traced on day $t$ of the simulation. We assume that there is a fixed lag in tracing, of $l$ days, and a base probability $\Theta$ of being considered for tracing in the first place (this includes things like certain contacts being non-traceable due to anonymity, the tracing system not being completely accurate, traced individuals not following regulations, etc.). Then, $\theta_{t,\tau}$ is calculated by the following multi-step process:

First, we calculate the probability that the infectious person whom the contact was with, was on day $x$ of their infectious period on the day of contact (i.e on day $t-\tau$):

$$\mathbb{P}_I(x) = \frac{I^D_{t-\tau,x}}{\sum_{\tau_I} I^D_{t-\tau,\tau_I}} \tag{28}$$

For the susceptible/exposed person to be traced on day $t$, the infectious person has to not have recovered by, and be detected on day $t-l$, or $\tau-l$ days after the contact occurred. If the infectious person was on day $x$ of their infectious period on the day of contact, they must therefore be on day $x+\tau-l$ of their infectious period when they were detected. Thus, the probability that the person was traced on day $t$, $\tau$ days after contact, **given** that the infectious person was on day $x$ of their infectious period on the day of contact, is given by:

$$\mathbb{P}_T(\tau|x) = \begin{cases} \dfrac{f_\delta(x+\tau-l)}{\sum_{\tau_I=x}^{21} f_\delta(\tau_I)} * \dfrac{\sum_{\tau_I=x+\tau-l}^{21} f_g(\tau_I)}{\sum_{\tau_I=x}^{21} f_g(\tau_I)} & \text{if } x+\tau-l \leq 21 \\ \\ 0 & \text{otherwise} \end{cases} \tag{29}$$

where $f_\delta$ is the pmf of the day of detection, discussed in the Detection Probabilities section, and $f_g$ is the pmf of the infectious period, discussed in the Infectious Period section.
Finally, the probabilities we need are given by:

$$\theta_{t,\tau} = \Theta \sum_{x=1}^{21} \mathbb{P}_I(x)\mathbb{P}_T(\tau|x) \tag{30}$$

## Contact Matrices

### Household

The household contact matrix is based on the household matrices from the POLYMOD [23, 24] and COVIMOD [25]studies. We assumed that care home residents had no household contacts (we use a separate care home matrix), and found from data from the German survey panel HaBIDS [26] that individuals in the MSM community had 0.48 times the household contacts with children as the non-MSM community. Apart from this, we divided contacts among the metapopulations proportional to the metapopulation size.

### Kindergarten

For children-teacher contacts in kindergarten, we used the results from a sensor-based study in Canada [27]. We assume that teacher-teacher contacts are not close enough to enable mpox transmission, and distribute contacts according to metapopulation size.

### Care

We use data from Asgary et. al. [28] to parametrise contacts in care homes, assuming that all workers are nurses. Once again, we assume that worker-worker contacts are not close enough to enable transmission, and distribute contacts accordingly. We also ignore visitor contacts.

### Sexual

To parametrise the number of sexual contacts in a day, we first obtained a 'number of contacts in a week' matrix from existing literature [29, 30]. To do this, we considered six sexual activity/preference classes: MSM(exclusively), MSM(bisexual), Non-MSM men, WSW(exclusively), WSW(bisexual), and Non-WSW women.
We used the method described by Walker et. al. [31] to create mixing matrices based on the age groups and these classes. Mixing was assumed to be 90% assortative in the non-MSM groups and 50% assortative in the MSM groups, while the classes were chosen proportionally given that a partnership was possible.
We combined this mixing matrix with the mean number of sexual partnerships in a year to obtain the 'weekly number of contacts' matrix. We then condensed the classes into 'MSM' and 'non-MSM', distributed partnerships among the various metapopulations, and divided the resulting matrix by 7 to obtain an approximation to 'mean number of sexual contacts in a day'.
Finally, to incorporate a 'high activity MSM class' in the sexual matrix to better model transmission pathways, we assumed that 20% of the MSM population were in this class, and that mean sexual contacts within this class (i.e. high activity-high activity sexual contacts) were $x$ times higher than those within the 'low activity' MSM class.

# 5 Metapopulation Distribution

Table S1.4 summarises the sources we used to try and make the metapopulation distribution used in the model as similar to the demographics in Berlin as possible (demographic distribution sources provide Germany-wide data; we generalised this to Berlin):

| **Data** | **Source** |
|---|---|
| Age and sex distribution | [32] |
| Number of children in Kindergarten | [33] |
| Proportion of male population that is MSM | [34] |
| Number of kindergarten teachers | [35] |
| Number of long-term care residents | [36] |
| Number of staff in long-term care | [37, 38] |

Table S1.4: Sources for demographic details of model
