# Supplementary material for: Estimating the relative importance of epidemiological and behavioural parameters for epidemic mpox transmission: a modelling study
Source: BMC Med. 2024 Jul 18;22:297. doi: 10.1186/s12916-024-03515-8 (PMC11256368; doi:10.1186/s12916-024-03515-8)
Supplement: Supplementary file 2 — Supplementary Material 2: Calibration and Validation of Model, Figs. S2.1–S2.3, Tables S2.1–S2.4. Fig. S2.1: Trace and density plots for Markov Chain Monte Carlo run. Fig. S2.2: Fitted model trajectories plotted against data used for fit. Fig. S2.3: Fitted model trajectories plotted against data not used for fit. Table S2.1: Bounds on parameters to be calibrated. Table S2.2: Initial values and proposal matrix variances for Markov Chain Monte Carlo run. Table S2.3: Labels for parameters used in figures. Table S2.4: Mean post-calibration values of parameters as used in the model. [file 12916_2024_3515_MOESM2_ESM.docx]

**Estimating the relative importance of epidemiological and behavioural parameters for epidemic mpox transmission: a modelling study**

Madhav Chaturvedi^1^, Isti Rodiah^2^, Mirjam Kretzschmar^3^, Stefan Scholz^4^, Berit Lange^2,5^, André Karch*^1^, Veronika K Jaeger*^1^

^1^Institute of Epidemiology and Social Medicine, University of Münster, Münster, Germany
^2^Department of Epidemiology, Helmholtz Centre for Infection Research, Braunschweig, Germany
^3^Department of Epidemiology, University Medical Center Utrecht, Utrecht, the Netherlands
^4^Martin-Luther-University Halle-Wittenberg, Halle, Germany
^5^German Center for Infection Research, Braunschweig, Germany

*contributed equally as last authors

**Supplementary File 2: Details of model calibration and validation**

**Methods**

1. *Calibration method*

We ran an adaptive particle Markov Chain Monte Carlo (pMCMC) algorithm using the R package mcstate version 0.9.18 [45] to sample from the posterior distribution of the parameters that were to be calibrated.

1. *Data used for calibration*

We fit the model to data about reported cases of mpox per calendar week (for calendar weeks 21-45) in Berlin, obtained from the Robert Koch institute database SurvStat [46]. We initialised our model at the start of calendar week 19.

1. *Parameters to be calibrated*

| **Parameter to be fitted** | **Bounds** |
| --- | --- |
| Probability of transmission per non-sexual contact | (0.0, 0.1) |
| Probability of transmission per sexual contact | (0.0, 0.5) |
| Proportion of infections that are detectable | (0.5, 1.0) |
| Initial number of infections (per age group in which infections initialised) (integer) | (0, ∞) |
| Ratio of sexual contacts of core MSM group to rest of MSM population | (10.0, 30.0) |
| Assortativity in sexual mixing between core group and rest of MSM population | (0.5, 1.0) |
| Probability that vaccination confers immunity* | (0.5, 1.0) |
| Base probability that a contact of a detected case will be traced | (0.5, 1.0) |

**Table S2.1:** Parameters that were calibrated to data, and bounds within which they were constrained.

1. *Parameter transformations*

To improve mixing of the pMCMC run, we allowed the algorithm to sample from the entire real line, and then transformed the parameters to satisfy the constraints required by the model and bounds in Table S2.1 before they were used in the model.

For the initial number of infections, a sampled real number $x$ was transformed into a positive integer by the transformation $\boldsymbol{f(x)=}\left\lfloor\left| \boldsymbol{x} \right| \right\rfloor$ i.e., the absolute value of $x$, rounded down to the nearest integer.

For all other parameters, a sampled real number $x$ was transformed to satisfy bounds $(a,b)$ via the transformation

$\boldsymbol{g}\left( \boldsymbol{x} \right)\boldsymbol{=a+}\frac{\boldsymbol{b-a}}{\boldsymbol{1+}\boldsymbol{e}^{\boldsymbol{-x}}}$

This is valid since the function $\boldsymbol{h: x}\underset{\to}{} \frac{\boldsymbol{1}}{\boldsymbol{1+}\boldsymbol{e}^{\boldsymbol{-x}}}$ is a continuous, monotonic bijection from the real line to the open unit interval $(0,1)$.

1. *Prior Distributions*

Uniform priors on the entire real line were used for all parameters.

1. *Likelihood and Particle Filter*

We assumed that the observed data followed a Poisson process with a mean equal to the cases detected in a week in the model (with a small amount of noise added to avoid zero expectation), and used a particle filter with 72 particles to compute the likelihood of the entire trajectory generated by a particular parameter proposal. It should be noted that, if the observed data is overdispersed, the Poisson distribution would lead to smaller-than-appropriate standard errors, and a negative binomial distribution would be more appropriate. However, since we only use the means of the posterior distributions in our analyses and theses are consistent across both distributions, we decided to reduce complexity by sticking to the Poisson distribution.

1. *Seed run*

To ensure that the pMCMC chain reached an area of high likelihood density within the proposal space, we first ran a seed chain of 500 steps. We set the proposal matrix to be a diagonal matrix consisting of the variances of each parameter shown in Table S2.2. The initial values for each parameter are also shown, both as used in the pMCMC (i.e., on the real line) and transformed.

| **Parameter** | **Initial value for pMCMC** | **Initial value, transformed for use in model** | **Variance (in untransformed value) for diagonal proposal matrix** |
| --- | --- | --- | --- |
| Probability of transmission per non-sexual contact | $h^{-1}(0.04)$ | 0.04 | 0.1 |
| Probability of transmission per sexual contact | $h^{-1}(0.1)$ | 0.1 | 0.1 |
| Proportion of infections that are detectable | $h^{-1}(0.8)$ | 0.9 | 0.1 |
| Initial number of infections (per age group in which infections initialised) | 10 | 10 | 2 |
| Ratio of sexual contacts of core MSM group to rest of MSM population | $h^{-1}(0.5)$ | 20 | 0.1 |
| Assortativity in sexual mixing between core group and rest of MSM population | $h^{-1}(0.9)$ | 0.95 | 0.1 |
| Probability that vaccination confers immunity | $h^{-1}(0.5)$ | 0.75 | 0.1 |
| Base probability that a contact of a detected case will be traced | $h^{-1}(0.65)$ | 0.825 | 0.1 |

Table S2.2: Initial values and proposal matrix variances for parameters for seed pMCMC run

1. *Main (adaptive) run*

After the seed run, we started the main, 5000-step pMCMC chain. The initial values for the parameters were the values in the last step of the seed run, and the initial proposal matrix was set to be the covariance matrix of the parameter samples from the seed run. In the main run, the proposal matrix was adapted over time; every 100 steps, the new proposal matrix was set to be the covariance matrix of the last 100 parameter samples.

Note: The adaptation of the proposal matrix was done manually since the adaptive pMCMC function provided in mcstate does not admit stochastic models.

*9. Model Validation*

We validated the model against reported cases of mpox per calendar week (for calendar weeks 25-37) in Cologne, downloaded from Survstat [46]**.** Some caveats must be mentioned here. First, in the absence of detailed demography data for the city of Cologne, we obtained a metapopulation distribution for Cologne by scaling down the metapopulation distribution for Berlin. Secondly, it is possible that the behaviour of the core group with high sexual contact frequency in Cologne may differ from the behaviour of the core group in Berlin, so behavioural parameters calibrated using data from Berlin may not be valid for Cologne.

**Results**

Table S2.3 provides a key for what the parameters are labelled as in the trace and density plots below.

| **Parameter** | **Label** |
| --- | --- |
| Probability of transmission per non-sexual contact | beta |
| Probability of transmission per sexual contact | beta_s |
| Proportion of infections that are detectable | detprop |
| Initial number of infections (per age group in which infections initialised) | init_I |
| Ratio of sexual contacts of core MSM group to rest of MSM population | increase |
| Assortativity in sexual mixing between core group and rest of MSM population | assort |
| Probability that vaccination confers immunity* | vacceff |
| Base probability that a contact of a detected case will be traced | traceprobbase |

Table S2.3: Labels for parameters in Figure S2.1

Figure S2.1 shows the trace and density plots for the main pMCMC run, with transformed parameters as used in the model, created using the coda package [47], version 0.19.4.

**Figure S2.1:** Trace and density plots for main pMCMC run, with parameters as used in the model.


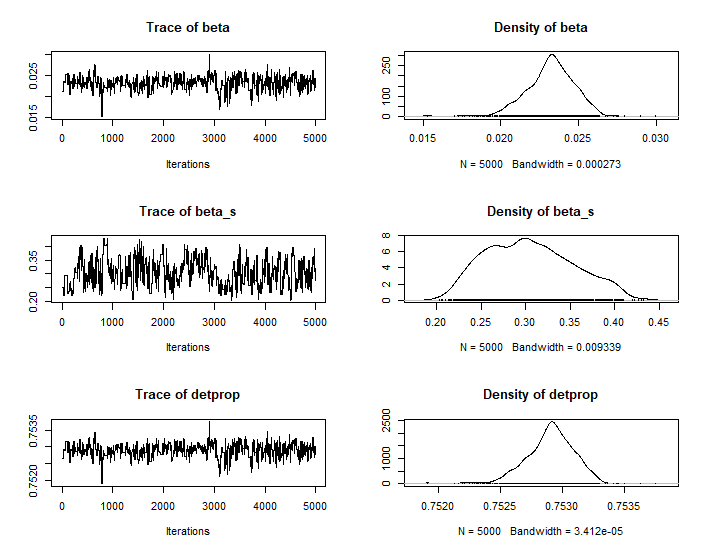

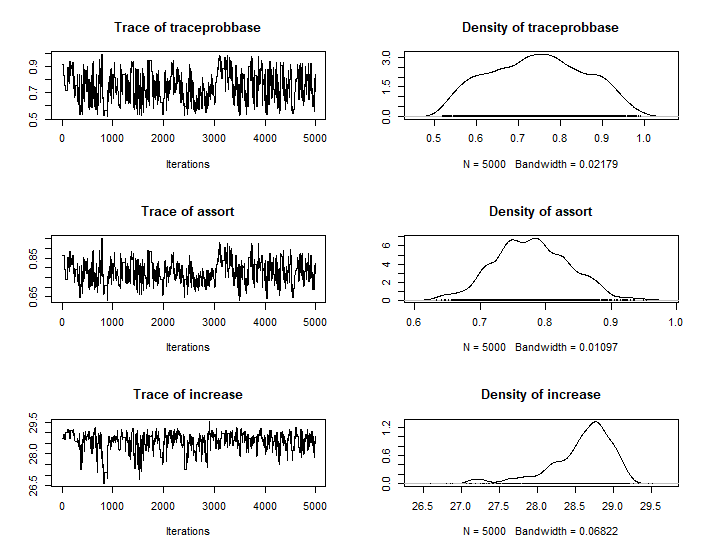

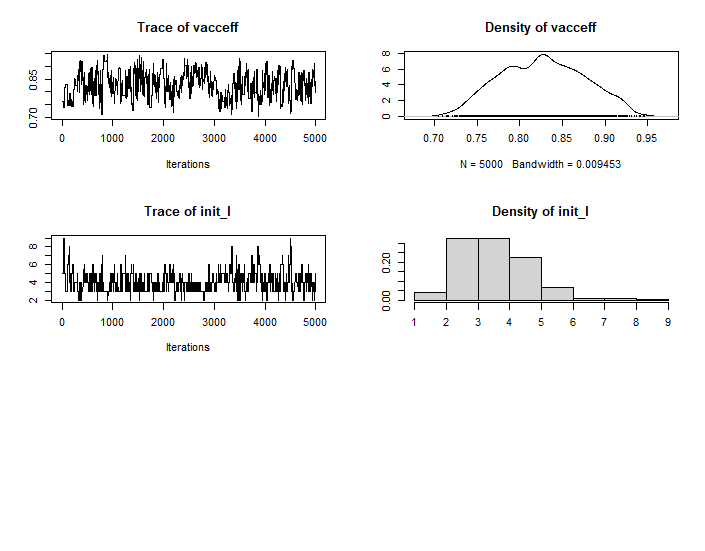


We took the means of the transformed parameters as the calibrated values for use in analyses. These are displayed in Table S2.4.

| **Parameter** | **Calibrated value** |
| --- | --- |
| Probability of transmission per non-sexual contact | 0.02 |
| Probability of transmission per sexual contact | 0.39 |
| Proportion of infections that are detectable | 0.75 |
| Initial number of infections (per age group in which infections initialised) | 4.03, rounded to 4 |
| Ratio of sexual contacts of core MSM group to rest of MSM population | 28.58 |
| Assortativity in sexual mixing between core group and rest of MSM population | 0.78 |
| Probability that vaccination confers immunity* | 0.83 |
| Base probability that a contact of a detected case will be traced | 0.75 |

Table S2.4: Mean values of transformed parameters sampled by the main pMCMC run

As a visual indicator of model fit, Figure S2.2 shows the number of observed cases and (particle-filtered) model trajectories using the fitted parameters, plotted against data about detected mpox cases in Berlin (i.e. the data used for calibration).


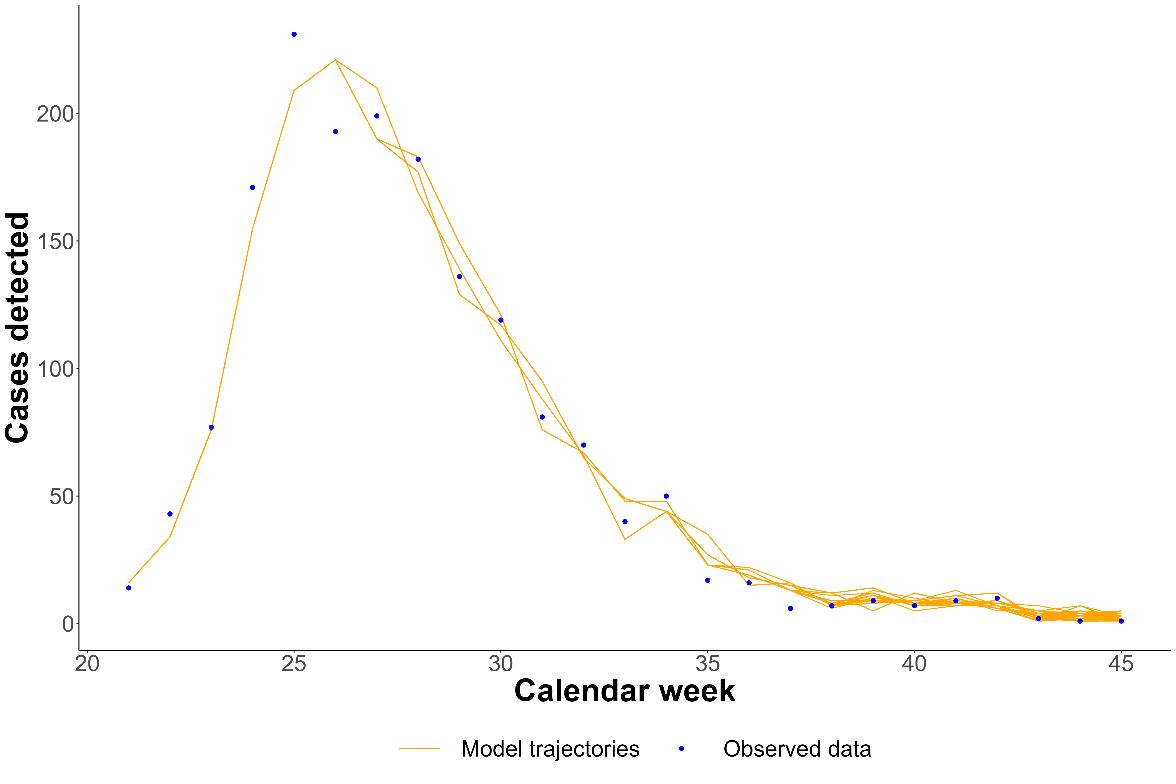


**Figure S2.2**: Observed data from Berlin and model trajectories with fitted parameters of mpox cases detected per calendar week.

Finally, as a visual indicator for model validation, Figure S2.3. shows (particle-filtered) model trajectories using the fitted parameters plotted against data about detected mpox cases in Cologne.


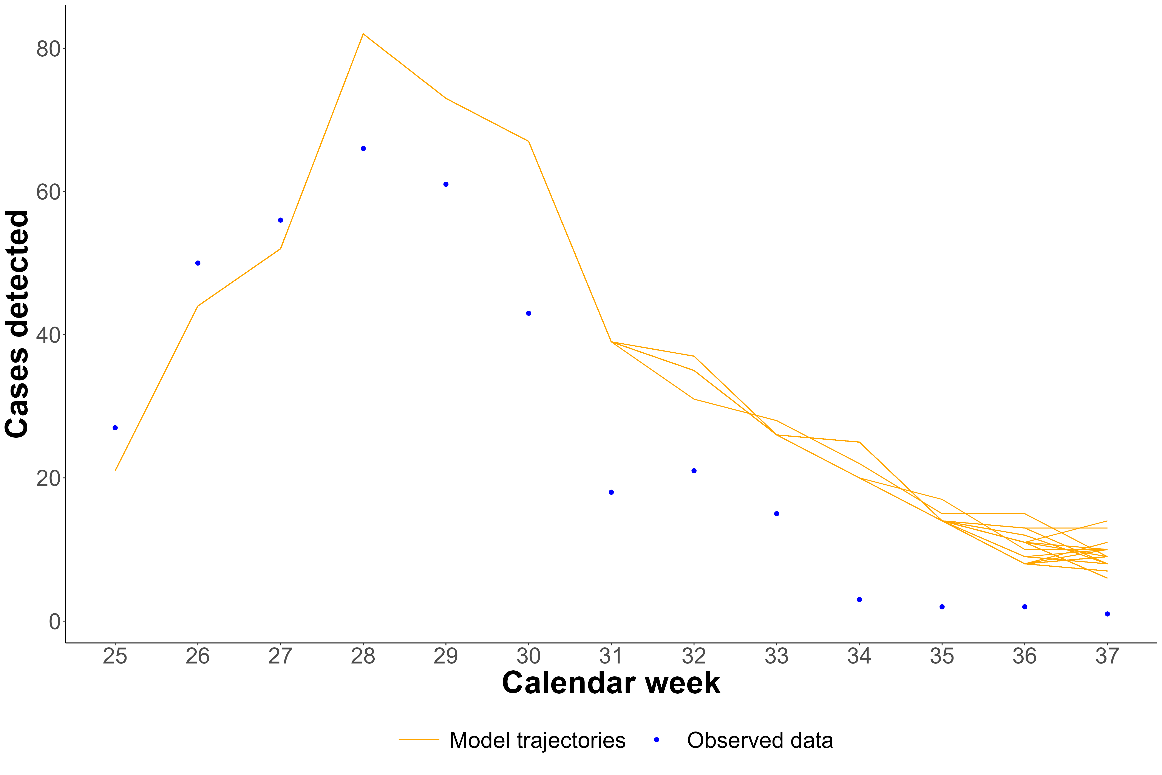


**Figure S2.3.:** Particle-filtered post-fitting model trajectories plotted against data about detected mpox cases per calendar week in Cologne.
